# AN INTERNATIONAL PERSPECTIVE ON OPTIMIZING FUNCTION AND ACTIVITY IN GERIATRIC CLIENTS ACROSS ALL SITES OF CARE

**DOI:** 10.1093/geroni/igad104.1116

**Published:** 2023-12-21

**Authors:** Sandra Zwakhalen, Silke Metzelthin, Barbara Resnick

## Abstract

With an increasing age, people are at risk for functional decline and care dependency. Frail older people in any site of care –at home, in nursing homes and in acute care– are supported by nursing staff to optimize their functioning, remain active, and maximize their independence. However, nursing staff often tend to work in a task-oriented manner and take over tasks unnecessarily which may deprive older people’s remaining abilities and adversely impact their life quality. Nursing staff require support to improve their activity supportive behavior in practice. This symposium presents the results of several international studies from primary care, home care, nursing home care and acute care, which aim to improve nursing staff behavior and –in turn– client outcomes. First, Prof. Galik will present the results of a randomized controlled theoretically-based trial testing the ‘Function-Focused Care’ approach in which over 350 patients participated. Dr. Vluggen will present the results of a cluster-randomized trial conducted in Dutch nursing home care in which the effectiveness of the ‘SELF-program’ is evaluated, including over 250 nursing staff and 200 patients. Dr. Metzelthin will discuss the feasibility of the SELF-program, which has been subjected to a thorough pilot-study in Dutch home care. Last, Prof. Graff will discuss the experiences of people with dementia and caregivers after following a coaching-based psychosocial program for primary care (SOCAV). The symposium is chaired by Dr. Vluggen and discussed by Prof. Resnick who has a considerable track record in intervention studies focusing on optimizing function and activity in geriatric clients.

